# Calculation of Heat Capacity Changes in Enzyme Catalysis and Ligand Binding

**DOI:** 10.1021/acs.jctc.2c00646

**Published:** 2022-09-12

**Authors:** Johan Åqvist, Florian van der Ent

**Affiliations:** Department of Cell & Molecular Biology, Uppsala University, Biomedical Center, SE-751 24 Uppsala, Sweden

## Abstract

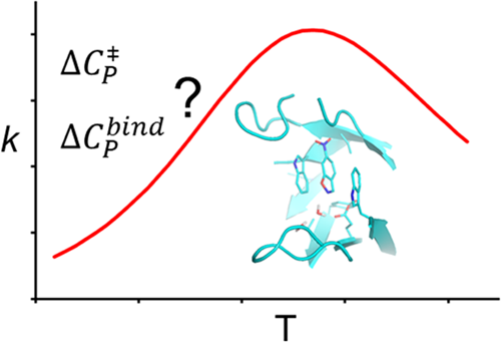

It has been suggested that heat capacity changes in enzyme catalysis may be the underlying reason for temperature optima that are not related to unfolding of the enzyme. If this were to be a common phenomenon, it would have major implications for our interpretation of enzyme kinetics. In most cases, the support for the possible existence of a nonzero (negative) activation heat capacity, however, only relies on fitting such a kinetic model to experimental data. It is therefore of fundamental interest to try to use computer simulations to address this issue. One way is simply to calculate the temperature dependence of the activation free energy and determine whether the relationship is linear or not. An alternative approach is to calculate the absolute heat capacities of the reactant and transition states from plain molecular dynamics simulations using either the temperature derivative or fluctuation formula for the enthalpy. Here, we examine these different approaches for a designer enzyme with a temperature optimum that is not caused by unfolding. Benchmark calculations for the heat capacity of liquid water are first carried out using different thermostats. It is shown that the derivative formula for the heat capacity is generally the most robust and insensitive to the thermostat used and its parameters. The enzyme calculations using this method give results in agreement with direct calculations of activation free energies and show no sign of a negative activation heat capacity. We also provide a simple scheme for the calculation of binding heat capacity changes, which is of clear interest in ligand design, and demonstrate it for substrate binding to the designer enzyme. Neither in that case do the simulations predict any negative heat capacity change.

## Introduction

**Figure 1:**
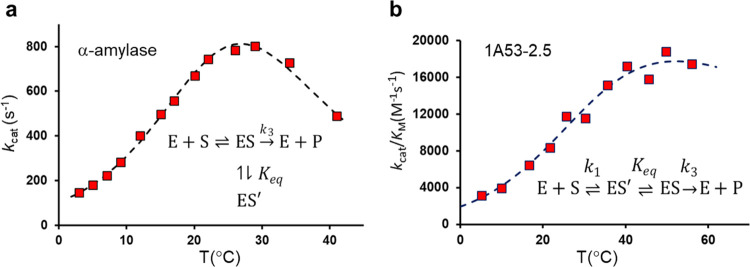
(a) Experimental *k*_cat_ versus temperature for the psychrophilic α-amylase,^10^ which was shown to be caused by an equilibrium with the inactive state ES′.^6^ This equilibrium is characterized by Δ*H*_eq_(ES′ – ES) ∼ 30 kcal/mol and Δ*S*_eq_(ES′ – ES) ∼ 0.11 kcal/mol/K and reflects breaking of an ionic interaction with the carbohydrate substrate at the *T*-optimum. The experimental data can also be fitted to eq 1, yielding a large negative Δ*C*_*p*_^‡^ = −0.98 kcal/mol/K, but such a model is incorrect in this case.^6^ (b) Experimental *k*_cat_/*K*_M_ versus *T* for the Kemp eliminase. The ES′ state in the kinetic scheme was again identified by computer simulations.^8^ This data can be fitted either with a Δ*C*_*p*_^‡^ = −0.3 kcal/mol/K (relative to either ES, ES′, or E + S) or Δ*C*_*p*_^bind^ = −0.3 kcal/mol/K or by all Δ*C*_*p*_’s zero and a change of rate-limiting step from *k*_1_ to *k*_3_ at 35 °C.^8^

The hypothesis that a nonzero activation heat capacity may underlie observations of curved Arrhenius plots in enzyme catalysis has recently been proposed in several studies.^1−5^ Particularly, if the rate-limiting transition state has a smaller heat capacity than the reactant state (Δ*C*_*p*_^‡^ < 0), this would induce a convex rate plot (with a maximum) due to the temperature-dependent activation enthalpies and entropies. Hence, the corresponding activation free energy becomes concave as a function of temperature, rather than linear, according to
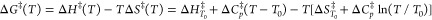
1where *T*_0_ is an arbitrary reference temperature (usually taken as 25 °C) and Δ*C*_*p*_^‡^ is assumed to be constant over the relevant temperature range. Such a model has thus been invoked to explain temperature optima in enzymes that are not related to the onset of protein unfolding.^1−5^ However, as we and others have shown earlier, there are other and maybe more intuitive explanations for such temperature optima and the curved Arrhenius plots associated with them.^6−9^ In the case of psychrophilic α-amylase from Antarctic bacterium *Pseudomonas haloplanktis*, the experimentally observed temperature optimum^10^ for *k*_cat_ and nonlinear Arrhenius plot could be directly captured by computer simulations of the catalytic reaction, which evaluated Δ*G*^‡^ as a function of temperature.^6^ Here, the optimum could be explained in terms of an increased population of an inactive state of the ES complex at higher temperatures (ES′ in Figure 1a), which is associated with breaking of a particular enzyme–substrate interaction.^6,7^ Hence, the emergence of inactive states along the reaction pathway can be one underlying cause of curved Arrhenius plots.

**Figure 2:**
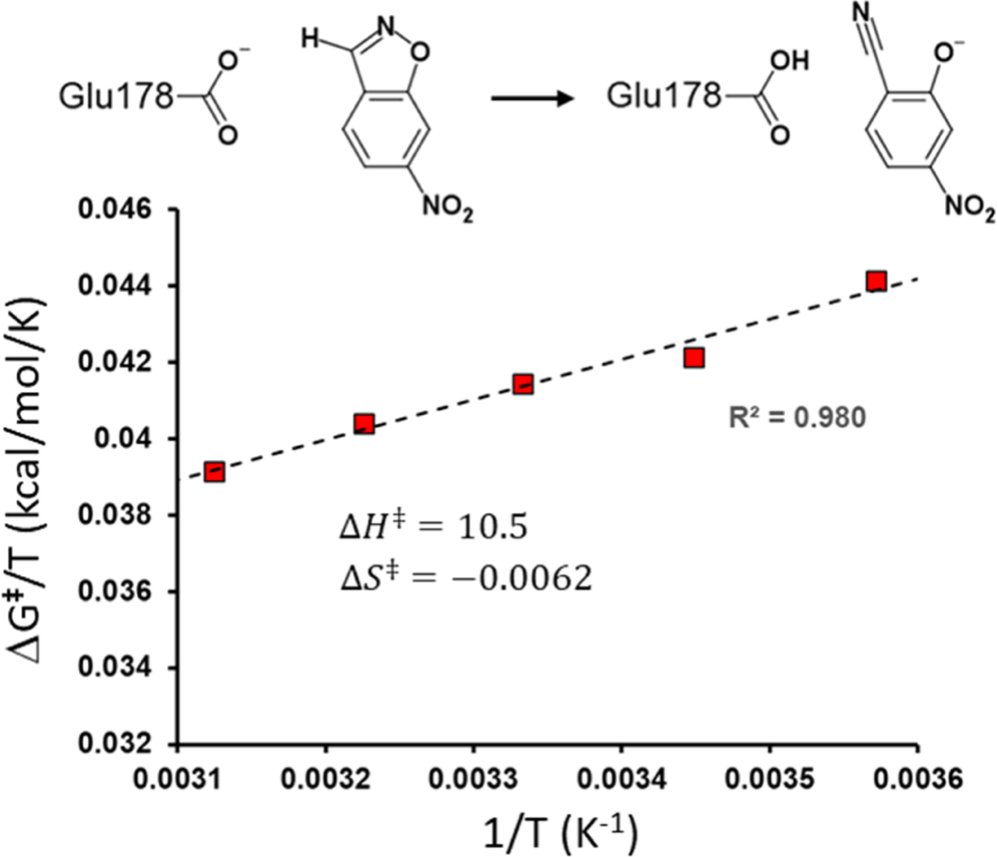
Reaction scheme and calculated Arrhenius plot of Δ*G*^‡^/*T* versus 1/*T* for the chemical step (*k*_3_) in the 1A53-2.5 Kemp eliminase.^8^

Another case in point is the 1A53-2.5 designer enzyme, optimized by directed evolution to catalyze a prototypic Kemp elimination reaction with the 6-nitrobenzisoxazole substrate. This enzyme shows a *k*_cat_/*K*_M_ rate optimum (Figure 1b) with a curved Arrhenius plot, and this was suggested by the authors to originate from *k*_cat_.^4^ It is somewhat unusual to analyze the composite rate constant *k*_cat_/*K*_M_ in terms of Arrhenius plots since it corresponds to at least two kinetic steps, but in the case of 1A53-2.5, the temperature dependence of *k*_cat_ could not be measured due to limited substrate solubility.^4^ Computer simulations of the chemical reaction step in 1A53-2.5 (*k*_3_ in Figure 1b), however, yielded a completely straight Arrhenius plot with *R*^2^ = 0.98 (Figure 2).^8^ Moreover, free energy calculations revealed the existence of a relaxed reactant state (ES′), with the reactants further apart in the active site, and the calculated van’t Hoff plot for the ES′ → ES transition was also linear to a good approximation (*R*^2^ = 0.89). It was found that ES lies about 3 kcal/mol above ES′ and no high free energy barriers separate the two states.^8^ These findings thus suggest that the observed behavior of *k*_cat_/*K*_M_ is not due to *k*_cat_ but must rather originate from the binding step. Here, two different explanations were found to be possible, either a change of the rate-limiting step from binding (*k*_1_) to chemistry (*k*_3_) at 35 °C or, simply, a heat capacity change upon substrate binding of Δ*C*_*p*_^bind^ = −0.3 kcal/mol/K.^8^ Although a binding heat capacity change of this magnitude is similar to that measured for inhibitor binding to some other enzymes,^11−13^ it was argued^8^ that the former explanation might be favored since the slower predecessor enzyme 1A53-2, with a very similar binding site, showed a straight plot for *k*_cat_/*K*_M_ with no sign of a nonzero Δ*C*_*p*_^bind^.^4^

In view of the considerable interest in understanding the origin of anomalous enzyme temperature optima of the type discussed above and, particularly, to assess the possibility of nonzero activation heat capacities, it is clearly paramount to search for reliable ways to estimate Δ*C*_*p*_^‡^ from computer simulations. There are basically two main routes to this problem. The first is to directly evaluate Δ*G*^‡^ (*T*) for a series of temperature points and determine whether Δ*G*^‡^ versus *T* or Δ*G*^‡^/*T* versus 1/*T* is linear or not (the latter type of Arrhenius plot is usually preferable since *R*^2^ then is more informative). This is the approach we have taken in evaluating enzyme activation parameters, in general, using molecular dynamics (MD) free energy calculations in combination with an empirical valence bond (EVB) description of the reaction potential energy surface (Figure 2).^14−16^ Since the accuracy of the Δ*G*^‡^ evaluations is critical and requires a large number of free energy calculations, MD/EVB is the method of choice here as it allows for very extensive configurational sampling that cannot at present be achieved by sufficiently accurate QM/MM methods. The direct calculation of Δ*G*^‡^(*T*) is, of course, also preferable since possible temperature optima will immediately be revealed as minima in Δ*G*^‡^(*T*) and allow direct estimation of the reaction rate constant from transition state theory.

The second approach does not actually attempt to calculate any activation free energies but just focuses on estimating Δ*C*_*p*_^‡^. If one has a reasonable force field model of the transition state of the reaction, then two MD simulations at a given temperature, of the reactant and transition states, can in principle suffice for determining whether they show any difference in heat capacity. Here, one would then use either of the two standard equations for the heat capacity, namely,
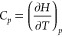
2or
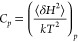
3where ⟨δ*H*^2^⟩ denotes the mean square fluctuation of the enthalpy. The enthalpy is usually replaced by the average total energy ⟨*E*_tot_⟩, and the difference between *C_p_* for the two systems, using either of the two equations, is then taken. Already the fact that eq 2 requires a converged value of ⟨*E*_tot_⟩ and eq 3 of ⟨δ*E*_tot_^2^⟩ would suggest that the former is more reliable for obtaining accurate estimates of *C*_*p*_, within a given simulation time span. Then, there is the additional problem with eq 3 of ensuring that MD simulations yield the correct total energy fluctuations, or energy distribution, and this is strongly dependent on the thermostat used in the MD simulations.^17,18^

Here, we revisit the case of the Kemp eliminase 1A53-2.5 and compare the different approaches for estimating Δ*C*_*p*_^‡^. We start by presenting some relevant benchmarks for liquid TIP3P water that address the influence of thermostats and their parameters, boundary conditions, etc. The results clearly show that the derivative formula is generally more reliable, and we also outline its use for obtaining estimates of possible heat capacity changes upon ligand binding to proteins.

## Methods

### Liquid Water Simulations

MD simulations were carried out with Q^19,20^ (https://github.com/qusers) and GROMACS^21,22^ software packages using the TIP3P water model.^23^ The Q simulations were carried out both with spherical droplets of a diameter of 40 Å and in a cubic periodic box with a side of 31 Å, yielding systems with 1141 and 1000 water molecules, respectively. The spherical systems were subjected to radial and polarization boundary restraints according to the surface constrained all-atom solvent (SCAAS) model,^19,24^ and the local reaction field (LRF) multipole expansion method^25^ was used to treat long-range electrostatic interactions beyond a direct cutoff of 10 Å, except for two benchmarks that used a plain 10 Å cutoff with no long-range treatment (cf. Table 1). Two such benchmarks with a plain cutoff were also run with periodic boundary conditions at a constant pressure of 1 bar using a Monte Carlo barostat.^26^ The Berendsen,^27^ Nosé–Hoover,^28^ and Langevin^29^ thermostats were employed in the simulations with Q using different values of the temperature coupling parameter, τ_T_.

**Table 1:** Heat Capacity per Molecule of TIP3P Water at 300 K Calculated with Equations 2 and 3 for Different Thermostats (kcal/mol/K)^a^

thermostat	parameters	boundary				
Berendsen	τ = 10 fs	sphere (LRF)	0.0175	0.0175	0.0089	0.0156
	τ = 100 fs	sphere (LRF)	0.0175	0.0174	0.0040	0.0127
	τ = 100 fs	sphere (cutoff)	0.0176	0.0176	0.0046	0.0135
	τ = 100 fs	box (PME)	0.0190	0.0190	0.0043	0.0131
	τ = 100 fs	box (cutoff)	0.0199	0.0198	0.0045	0.0131
Nosé–Hoover	τ = 100 fs	sphere (LRF)	0.0175	0.0175	0.0174	0.0175
	τ = 100 fs	sphere (cutoff)	0.0177	0.0177	0.0173	0.0176
	τ = 100 fs	box (PME)	0.0190	0.0190	0.0196	0.0198
	τ = 1000 fs	box (PME)	0.0190	0.0191	0.0194	0.0194
	τ = 100 fs	box (cutoff)	0.0199	0.0199	0.0181	0.0185
Langevin	τ = 10 fs	sphere (LRF)	0.0180	0.0175	0.0190	0.0177
	τ = 100 fs	sphere (LRF)	0.0174	0.0173	0.0185	0.0181
	τ = 2000 fs	box (PME)	0.0193	0.0192	0.0205	0.0202
v-rescale	τ = 100 fs	box (PME)	0.0191	0.0190	0.0195	0.0194

a Convergence errors are in all cases ≤0.001 kcal/mol/K.

The GROMACS simulations were carried out with a periodic box of the same size as in Q and utilized the particle mesh Ewald (PME) method^30^ for treatment of long-range electrostatic interactions with a short-range cutoff of 10 Å. A Parrinello–Rahman barostat^31^ was used here with a pressure coupling constant of τ_P_ = 1 ps and a compressibility of 4.5 × 10^–5^ bar^–1^. MD simulations were run with the same thermostats as above and also with the velocity rescale algorithm of Bussi et al.^32^ In the GROMACS simulations, the temperature coupling was applied every ten MD steps, except for the Nosé–Hoover simulations with τ_T_ = 1 ps, in which case the coupling was applied every step. Both Q and GROMACS simulations employed a 2 fs time step, and production runs of 1 ns at each of five different temperatures (280, 290, 300, 310, and 320 K) were generated after 100 ps of initial equilibration.

### Enzyme Simulations

The enzyme MD simulations were run as described earlier^8^ starting from the equilibrated structures of ref (8). These used the crystallographic structure of 1A53-2.5 in complex with 6-nitrobenzotriazole (PDB entry 6NW4)^4^ as the starting point, where the inhibitor was changed to the 6-nitrobenzisoxazole substrate. The solvated spherical simulation systems (50 Å in diameter) were centered on the substrate C1 carbon, and protein atoms lying outside this sphere were tightly restrained to their crystallographic positions and excluded from nonbonded interactions (96% of the protein atoms are unrestrained inside the simulation sphere).^8^ The apo enzyme structure was simply modeled by removing the inhibitor from the 6NW4 structure, resolvated, and equilibrated as described earlier.^8^ All enzyme MD simulations were performed with the Q software package^19,20^ with a 1 fs time step utilizing the OPLS-AA/M force field,^33^ the LRF method^25^ for long-range electrostatics, and the SCAAS solvent boundary restraints.^19,24^ As earlier, a 10 kcal/mol/Å^2^ flat-bottom harmonic restraint was applied to the donor–acceptor (C···O) distance (>3.0 Å) in the reactant state to keep the donor and acceptor atoms in contact.^8^ We also set up one set of simulations without this restraint to examine the heat capacity of the relaxed reactant complex (ES′ in Figure 1b). The transition state of the chemical reaction was modeled with a two-state EVB potential that had fixed coefficients of *c*_1_^2^ = *c*_2_^2^ = 0.50 for the reactant and product states. This choice corresponds to the average values of the EVB coefficients in the TS ensemble.^8^ Sample input files for running the simulations are provided at the Zenodo repository with DOI: 10.5281/zenodo.7023187.

We thus carried out four sets of enzyme MD simulations for the apo enzyme, the relaxed reactant state (ES′), the contact reactant state (ES), and the transition state (TS). These were all run with the same settings as in ref (8) and used the Berendsen thermostat with a strong heat bath coupling of τ_T_ = 10 fs. For comparison, we additionally carried out the simulations of ES and TS with the Nosé–Hoover thermostat and a coupling of τ_T_ = 100 fs.

Each of these six sets of simulations consisted of 30 ns of data collection at each of the five temperatures (see above), yielding a total of 150 ns per enzyme heat capacity calculation.

## Results

### Liquid Water Simulations

To gauge the accuracy of eqs 2 and 3 in computing heat capacities, it is useful to first consider the standard benchmark of liquid water, represented here by the rigid TIP3P model.^23^ We thus set up a series of simple water simulations at five different temperatures using either spherical or periodic boundary conditions, with or without treatment of long-range electrostatics, and with different thermostats. These results are summarized in Table 1. For example, it is well known^17,18^ that the popular Berendsen thermostat^26^ does not yield canonical ensemble averages but rather something in between the canonical and microcanonical situations.^17^ In particular, its energy fluctuations are too small and become smaller with larger temperature relaxation time (τ_T_), which is the only parameter of the thermostat. However, the derivative formula (eq 2) gives consistent results and values of *C*_*p*_ per molecule of about 0.0175 and 0.0190 kcal/mol/K for the spherical and periodic systems, respectively, independent of the thermostat and τ_T_. These values are in good agreement with the experimental value^34^ of 0.0180 kcal/mol/K and clearly show that eq 2 gives robust results. Note that sometimes a correction for intramolecular vibrations is added to the calculated values when compared to experiment,^35^ but this does not really serve any purpose since the water model is what it is, namely, rigid. Taking only the average potential energy ⟨*U*_tot_⟩ per molecule in eq 2 plus a kinetic 3*R* correction for translation and rotation also yields identical results to those for ⟨*E*_tot_⟩. Moreover, inclusion of long-range electrostatic interactions, either by PME (periodic systems)^30^ or LRF (spherical systems),^25^ has a negligible effect on *C*_*p*_, although it can be seen to very slightly reduce the heat capacity (Table 1).

In contrast, the fluctuation formula of eq 3 with the Berendsen thermostat clearly yields *C*_*p*_ values that are much smaller than those from eq 2 or experiment. As expected, the deviation becomes larger when the value of τ_T_ is increased since coupling to the heat bath then becomes weaker. It may be noted that the ⟨δ*U*_tot_^2^⟩ + 3*R* approximation is better than ⟨δ*E*_tot_^2^⟩ but still in error, which reflects the fact that the kinetic energy fluctuations are more damped by the thermostat. The remedy needed for the use of eq 3 is then to change the thermostat, and, as can be seen from Table 1, this consistently raises the mean square fluctuations and *C*_*p*_ for the Nosé–Hoover,^28^ Langevin^29^ and velocity rescaling^32^ thermostats. However, it is evident that the derivative formula (eq 2) still gives more consistent results than eq 3 since the fluctuation formula is more sensitive to the parameters used.

Hence, the conclusion from the above benchmarks is that the derivative formula basically works and gives consistent results irrespective of the thermostat used, while the fluctuation formula requires careful attention to the thermostat and probably also longer simulations for good statistics. Actually, most studies that have calculated heat capacities for liquid systems have indeed also used the derivative formula.^35−37^ With regard to spherical versus periodic boundaries, one can also see that the former gives slightly lower values of *C*_*p*_ due to the finite system but is at least as close to the experimental value as the periodic.

### Enzyme Simulations

The question raised in the Introduction section was how we could estimate a possible nonzero value of Δ*C*_*p*_^‡^ in enzyme catalysis. The earlier calculated Arrhenius plot for the chemical transformation step in the designer Kemp eliminase 1A53-2.5 was found to be very close to linear, with constant values of Δ*H*^‡^ = 10.5 kcal/mol and Δ*S*^‡^ = −0.0062 kcal/mol/K, obtained from linear regression (Figure 2). Note that the underlying values of Δ*G*^‡^ come directly from MD/EVB reaction free energy profile calculations at 280, 290, 300, 310, and 320 K, with 30 replicate simulations carried out at each temperature for a total simulation time of about 160 ns.^8^ Any attempt to improve the linear fit by inserting the above values as initial guesses of Δ*H*_*T*_0__^‡^ and Δ*S*_*T*_0__^‡^ in eq 1 yields a negligibly small value of Δ*C*_*p*_^‡^ = 0.00016 kcal/mol/K. Hence, it is evident that the Arrhenius plot approach for estimating Δ*C*_*p*_^‡^ yields a value of zero for the chemical reaction step in the Kemp eliminase.

Interestingly, van der Kamp, Mulholland, and co-workers have attempted to use the fluctuation formula (eq 3) with ⟨δ*U*^2^⟩ to estimate Δ*C*_*p*_^‡^ both for the Kemp eliminase 1A53-2.5^5^ and two other enzymes,^3^ with force field models of the reactant (ES) and transition states (TS), and found negative values of Δ*C*_*p*_^‡^ from these calculations. These calculations used the Berendsen thermostat with a large τ_T_ of 10 ps, which, as shown above, severely underestimates the energy fluctuations. Moreover, all solvent except ten water molecules in the active site was removed from the calculations of ⟨δ*U*^2^⟩ in the 1A53-2.5 enzyme,^5^ and in the other two cases, all of the solvent was discarded.^3^ So, essentially these simulations measured only the protein and substrate energy fluctuations ⟨δ*U*_prot_^2^⟩ instead of ⟨δ*U*_tot_^2^⟩. For the Kemp eliminase, this resulted in a totally unrealistic value of Δ*C*_*p*_^‡^ = −4.7 kcal/mol/K for the chemical step with the reactants in contact (ES state), which does not fit the experimental data. That is, if the activation free energy associated with *k*_cat_/*K*_M_ (i.e., E + S → TS) derived from experiments^4^ is fitted according to eq 1, one obtains a value of Δ*C*_*p*_^‡^ = −0.3 kcal/mol/K. As discussed above, it is possible that this value simply reflects a negative binding heat capacity change (Δ*C*_*p*_^bind^ = −0.3 kcal/mol/K), which is embedded in *k*_cat_/*K*_M_ since this quantity reflects both the binding and chemical steps. As also mentioned, the other possibility suggested by our calculated straight Arrhenius plot for the chemical step is that there is a change in the rate-limiting step that would, in fact, cause a dip of the same magnitude in the Δ*C*_*p*_ value associated with 1/*K*_M_ at the temperature where this change occurs.^8^

**Figure 3:**
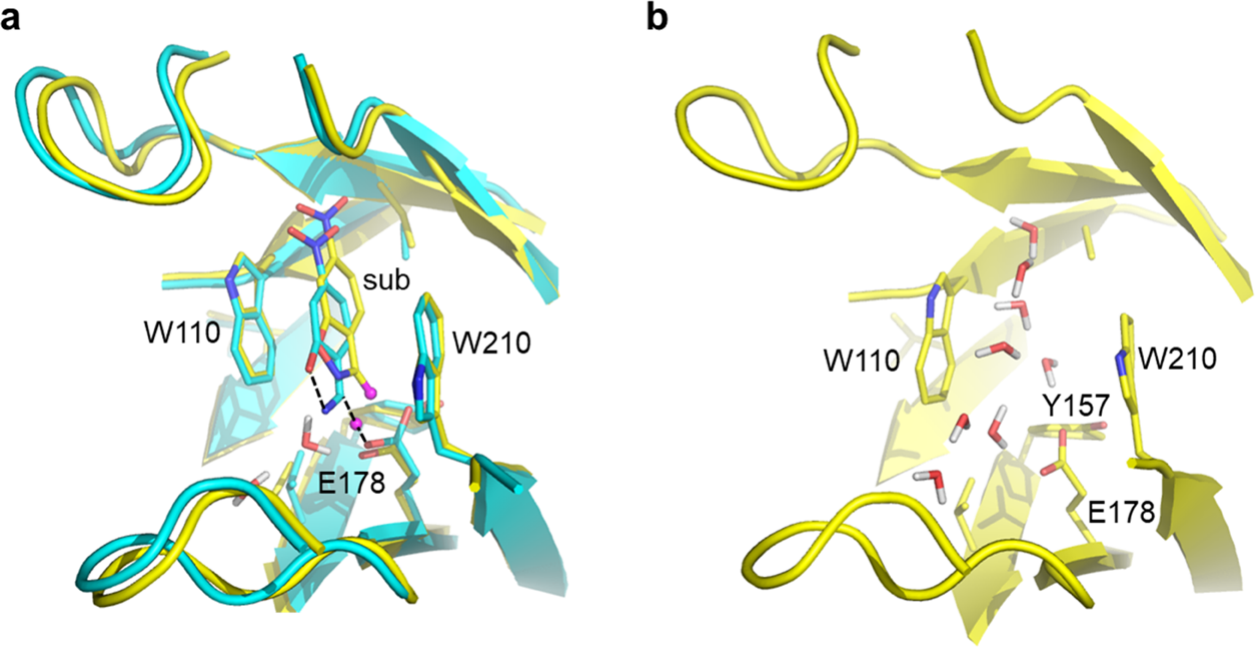
(a) Representative MD snapshots of the ES (yellow) and TS (cyan) structures used in the heat capacity calculations. Partially broken and formed bonds in the TS are denoted by dashed lines. (b) Model of the apo enzyme after 250 ps of MD equilibration, illustrating the entry of solvent replacing the substrate.

Clearly, it is of major interest to try to reconcile the results for Δ*C*_*p*_^‡^ calculations based on reaction free energy profiles and Arrhenius plots with those from plain MD simulations at the reactant and transition states. To this end, we first carried out MD simulations of the contact reactant state of 1A53-2.5 with the 6-nitrobenzisoxazole substrate (ES) and of the approximate transition state (TS) with fixed EVB coefficients of *c*_1_^2^ = *c*_2_^2^ = 0.50 (Figure 3a), where the two valence bond states correspond to the reactant and product.^8^ This choice of EVB coefficients corresponds to the average values of the TS ensemble^8^ but is an approximation since *c*_1_^2^ and *c*_2_^2^ fluctuate during actual MD/EVB simulations. Moreover, since the true EVB energy for any configuration is given by the solution of the 2 × 2 secular equation as^38^
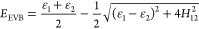
4the force field approximation 0.5 (ε_1_ + ε_2_) also lacks the influence of a fluctuating energy gap (ε_1_ – ε_2_) and the *H*_12_ off-diagonal coupling element on the calculated energy. The simulations were performed in exactly the same way as earlier using a 50 Å diameter spherical system with 96% of the enzyme moving freely and solvated by TIP3P water.^8^ A 10 kcal/mol/Å^2^ distance flat-bottom distance restraint (<3.0 Å) was applied between the donor and acceptor atoms involved in the proton transfer from the substrate (C1) to Glu178 (Oε1). This setup for the two states, including the Glu178-substrate restraint, is thus very similar to that used in ref (5). We again carried out MD simulations at five temperatures to examine both the derivative and fluctuation formulas for the heat capacities.

**Figure 4:**
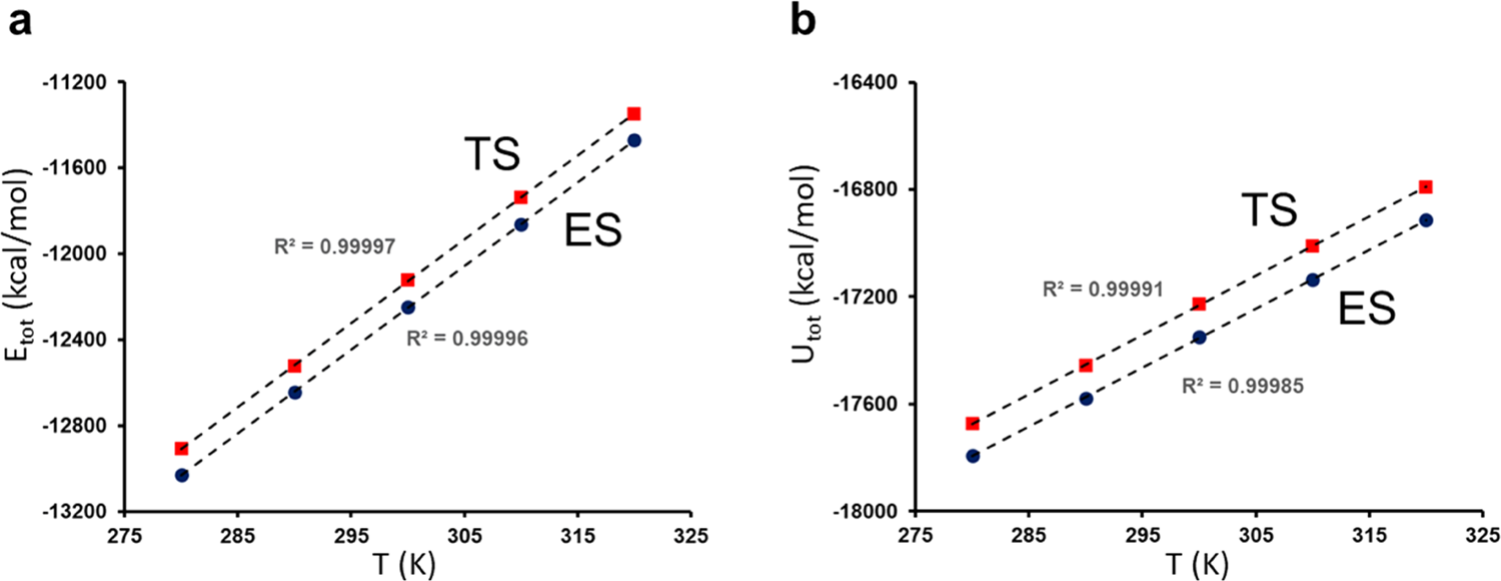
Plots of the average total energies (a) and total potential energies (b) for the reactant state (ES) and transition state (TS) from MD simulations.

Our standard simulation protocol uses the Berendsen thermostat with separate scaling of solute and solvent atoms and a strong heat bath coupling of τ_T_ = 10Δ*t* = 10 fs to have very well-defined temperatures. The derivative formula (eq 2) yields almost identical values of *C*_*p*_(ES) = 38.95 and *C*_*p*_(TS) = 39.05 kcal/mol/K (Table 2). Here, it is important to assess the magnitude of errors, and one measure is simply block averaging by splitting the data at each temperature into two blocks. This yields estimated errors of 0.08 and 0.09 kcal/mol/K (∼0.2%) for *C*_*p*_(ES) and *C*_*p*_(TS), respectively. An alternative error estimate is the asymptotic standard error of the linear regression yielding the derivative (Figure 4), and this measure gives somewhat larger errors of 0.14 and 0.13 kcal/mol/K (∼0.35%). The former error estimate is probably more informative of MD simulation convergence since the latter only reports on the quality of fit and may be affected by, e.g., a slight variation of *C*_*p*_ with temperature (both measures are given in Table 2). Note that the absolute *C*_*p*_ values for the solvated enzyme are large since they correspond to the entire system and are not scaled by the number of molecules as above in the liquid water simulations.

**Table 2:** Calculated Heat Capacities (kcal/mol/K) for the Different States in the 1A53-2.5 Enzyme with Equations 2 and 3^a^

						
Berendsen τ = 10 fs						
*C*_*p*_(ES)	38.95 (0.08)	38.93 (0.05)	27.28 (0.16)	14.32 (0.15)	34.42 (0.12)	68.67 (3.43)
	(0.14)	(0.16)	(0.53)			
*C*_*p*_(TS)	39.05 (0.09)	39.03 (0.07)	27.76 (0.32)	14.53 (0.16)	34.63 (0.14)	63.61 (1.63)
	(0.13)	(0.12)	(0.33)			
Δ*C*_*p*_^‡^(TS – ES)	**0.10** (0.12)	**0.10** (0.09)	**0.48** (0.36)	**0.21** (0.22)	**0.21** (0.18)	**–5.07** (3.80)
	(0.19)	(0.20)	(0.62)			
Nosé–Hoover τ = 100 fs						
*C*_*p*_(ES)	38.17 (0.03)	38.12 (0.03)	27.69 (0.52)	45.56 (0.53)	45.02 (0.57)	85.88 (4.38)
	(0.21)	(0.21)	(0.66)			
*C*_*p*_(TS)	38.32 (0.01)	38.26 (0.02)	27.96 (0.07)	45.33 (0.58)	44.70 (0.59)	83.72 (3.61)
	(0.12)	(0.12)	(0.74)			
Δ*C*_*p*_^‡^(TS – ES)	**0.15** (0.03)	**0.15** (0.04)	**0.27** (0.52)	**–0.33** (0.79)	**–0.32** (0.82)	**–2.15** (5.68)
	(0.25)	(0.25)	(0.99)			
Berendsen τ = 10 fs						
*C*_*p*_(ES′)	38.90 (0.06)	38.86 (0.04)				
	(0.18)	(0.19)				
*C*_*p*_(apo)	38.80 (0.07)	38.74 (0.06)				
	(0.13)	(0.13)				
Δ*C*_*p*_(wat – lig)	**–0.09** (0.02)	**–0.11** (0.02)				
	(0.19)	(0.18)				
Δ*C*_*p*_^bind^(ES′ – apo)	**0.00** (0.09)	**0.03** (0.07)				
	(0.29)	(0.29)				
Δ*C*_*p*_^‡^(TS – apo)	**0.16** (0.12)	**0.18** (0.09)				
	(0.26)	(0.25)				

a Errors are given for eq 2 in parentheses both from block averaging (first entry) and as the asymptotic standard error of the mean (s.e.m.) for the linear regression (second entry). Errors for eq 3 are given as the s.e.m. for all five temperatures. The entry Δ*C*_*p*_(wat – lig) corresponds to the difference in heat capacity between a sphere of pure water with a diameter of 40 Å and a sphere with the same number of water molecules that also contains one substrate molecule based on 1 ns simulations at each of the five temperatures. Bold face entries signify Δ*C*_*p*_ values (differences) as opposed to absolute *C*_*p*_ values.

We can thus estimate Δ*C*_*p*_^‡^ = 0.097 kcal/mol/K from eq 2 using ⟨*E*_tot_⟩ for the two states (ES and TS), and a very similar number is obtained with ⟨*U*_tot_⟩ (Table 2). The plots of ⟨*E*_tot_⟩ and ⟨*U*_tot_⟩ versus temperature are shown in Figure 4. These values of Δ*C*_*p*_^‡^ are thus slightly positive, rather than negative, but their magnitude is within our error bars. These results are therefore in agreement with the calculated Arrhenius plot, and both methods thus essentially yield a zero value of Δ*C*_*p*_^‡^. If we instead try to use the protein and substrate potential energies only, ⟨*U*_prot_⟩, and correct ∂*U*_prot_/∂*T* with the kinetic energy term *N*_df_*R*/2, where *N*_df_ is the number of degrees of freedom, the absolute heat capacities become much lower than the correct ones since no solvent interactions are included in *U*_prot_ (Table 2). With this approach, we still obtain a positive value of Δ*C*_*p*_^‡^ = 0.479 kcal/mol/K. However, now the estimated errors increase by a factor of 3 for the two states so that this value is still close to zero within our error bars.

As expected with the Berendsen thermostat, the fluctuation formula (eq 3) also gives much too low absolute values of *C*_*p*_ of about 14 kcal/mol/K for the two states when using ⟨δ*E*_tot_^2^⟩ (Table 2). The total potential energy fluctuations (when corrected by *N*_df_*R*/2) also yield a too low heat capacity but closer to the value from the derivative formula, which again reflects the fact that it is primarily the kinetic energy fluctuations that are damped by the thermostat (as in the water simulations above). Here, we report the standard errors of the mean (s.e.m.) for the *C*_*p*_ values calculated from all five temperatures (Table 2) to consider the same amount of data as in the derivative calculations. If one only retains the protein and substrate potential energy fluctuations, on the other hand, the absolute *C*_*p*_ estimates become much too large and are associated with very large errors. The fact that ⟨δ*U*_prot_^2^⟩/*kT*^2^ is significantly larger than ⟨δ*E*_tot_^2^⟩/*kT*^2^ or ⟨δ*U*_tot_^2^⟩/*kT*^2^ (the latter being ∼18 kcal/mol/K without the kinetic correction) clearly shows that there is major compensation (anticorrelation) going on between protein and solvent energies. Hence, the isolated ⟨δ*U*_prot_^2^⟩ component is not a meaningful quantity at all. If one takes the difference between the incorrect ⟨δ*E*_tot_^2^⟩ or ⟨δ*U*_tot_^2^⟩ values between TS and ES, this yields in both cases Δ*C*_*p*_^‡^ estimates of +0.21 kcal/mol/K, which is again zero within the now larger error bars than for the derivative formula. Taking this difference for only the protein component (⟨δ*U*_prot_^2^⟩) gives a completely unrealistic value of Δ*C*_*p*_^‡^ = −5.07 kcal/mol/K, associated with huge errors (Table 2).

Switching to the Nosé–Hoover thermostat in the enzyme simulations does not change the above picture very much, except for the fact that the absolute heat capacities calculated with the fluctuation formula now all increase as a consequence of the fluctuations now being generally larger. This is also reflected by an increase of the error bars (Table 2). The derivative formula now gives Δ*C*_*p*_^‡^ = 0.15 kcal/mol/K, which is very similar to the Berendsen thermostat. It may be noted that the absolute *C*_*p*_ values calculated from ⟨δ*E*_tot_^2^⟩ and ⟨δ*U*_tot_^2^⟩ with the Nosé–Hoover thermostat are now somewhat larger than those from the derivative formula, which suggests that the τ_T_ parameter of the thermostat should first be optimized or fine-tuned to make eqs 2 and 3 give identical results. The Δ*C*_*p*_^‡^ values calculated from these fluctuations are now slightly negative but again within the errors. Obviously, the derivative formula gives more reliable results with smaller errors, irrespective of the thermostat used.

### Estimating the Heat Capacity Change for Substrate Binding

The fact that the derivative formula gives very stable results, which are not particularly sensitive to the thermostat and its parameters, suggests that this equation could also be used to examine a possible heat capacity change upon substrate binding. We thus repeated the enzyme calculations for the apo enzyme without the bound substrate, which leads to some additional water molecules initially replacing the substrate (Figure 3b). In this case, we only consider the derivative formula (eq 2) in view of the above results and use the Berendsen thermostat, as employed in the earlier Arrhenius plots calculations (Figure 2).^8^ Moreover, since our earlier free energy calculations on releasing the donor–acceptor (C···O) distance restraint in the (contact) reactant state revealed the existence of the relaxed reactant state (ES′) that is a few kcal/mol lower in energy,^8^ we also repeated the *C*_*p*_ calculations for this state. The calculated *C*_*p*_ value for ES′ using eq 2 is 38.90 ± 0.06 kcal/mol/K (block averaging error), which is thus virtually identical to the value for ES.

When calculating the binding heat capacity, it is important to consider the proper thermodynamic process that, besides inserting the substrate into the enzyme active site, also involves removing it from liquid water. The two contributions can be evaluated separately and summed up as apo-enzyme + ligand-in-water → holo-enzyme + pure water. It is then essential that the total number of degrees of freedom on the two sides of the equation are balanced since each degree of freedom contributes to the absolute heat capacity. The way to achieve this is to consider the same number of water molecules in the apo and holo enzyme systems and similarly for the pure water and solvated ligand systems. The number of degrees of freedom for the two enzyme systems will then differ only by those of the ligand, which is balanced by the same difference for the two solution systems. The resulting Δ*C*_*p*_^bind^ will then be the difference between the partial molar heat capacities of the ligand in the enzyme and water.

As can be seen from Table 2, the calculated *C*_*p*_ values for the apo enzyme are also very similar to those obtained for reactant and transition states, all being about 39 kcal/mol/K for this simulation system. If one takes the differences Δ*C*_*p*_^bind^(ES′ – apo) and Δ*C*_*p*_^‡^(TS – apo), after adding the Δ*C*_*p*_(wat – lig) contribution of −0.09 kcal/mol/K, the former binding heat capacity is predicted to be 0.00 kcal/mol/K and the latter activation heat capacity is predicted to b +0.16 kcal/mol/K (which now corresponds to *k*_cat_/*K*_M_). Both of these quantities are thus again very close to zero (Table 2). Hence, we see no sign of any negative heat capacity differences either with regard to enzyme activation or substrate binding to the 1A53-2.5 Kemp eliminase. The rather accurate estimate of 0.10 ± 0.12 kcal/mol/K obtained for Δ*C*_*p*_^‡^(TS – ES) is essentially zero, in agreement with our calculated linear Arrhenius plot (Figure 1). Notably, this estimate differs significantly from a Δ*C*_*p*_^‡^ value of −0.3 kcal/mol/K that would be required if the experimentally observed curvature were to be explained by a *k*_cat_ effect.^4,8^ Furthermore, if Δ*C*_*p*_^bind^ is also close to zero, as it indeed appears to be, our earlier result from kinetic modeling that a change of rate-limiting step could be responsible for the curved *k*_cat_/*K*_M_ Arrhenius plots indeed appears as the most plausible explanation.^8^

## Discussion

We have shown here that the calculation of heat capacities from MD simulations by means of the derivative formula (eq 2), either in terms of the total energy or the potential energy, is generally more reliable than the fluctuation formula of eq 3. In particular, the latter equation fails severely with the Berendsen thermostat and becomes worse the longer the temperature relaxation time that is used. Also, in the case of enzyme simulations, the derivative formula clearly is the most robust one, irrespective of the thermostat. Moreover, in that case, the idea that only the protein part of the total potential energy would suffice for reliable heat capacity calculations with the fluctuation formula is clearly disproved here.

In general, accurate calculations of heat capacities for enzymes may be of considerable interest in enzymology since possible heat capacity differences associated either with substrate binding (Δ*C*_*p*_^bind^) or catalysis (Δ*C*_*p*_^‡^) could, in principle, give rise to nonlinear Arrhenius plots. That is, if Δ*C*_*p*_^bind^ < 0 for the binding event, this could yield a rate optimum for *k*_cat_/*K*_M_ that is unrelated to unfolding of the enzyme. Such negative binding heat capacities have indeed been observed in some cases for enzyme–inhibitor complexes, and the magnitude of Δ*C*_*p*_^bind^ then typically appears to be a few tenths of a kcal/mol/K.^11−13^ This is perhaps not so surprising since binding of a substrate or inhibitor could be considered somewhat analogous to folding of a protein if it induces a significant stabilization of the structure. What seems less intuitive is the idea that there could be a negative value of Δ*C*_*p*_^‡^ associated with the chemical step in the enzyme (*k*_cat_). That is, since a typical chemical transformation only involves the formation or breaking of a few bonds (e.g., Figure 3a), it is difficult to see how this could cause a change in the overall heat capacity of the system when the reaction barrier is climbed. However, if this were the case, it would indeed also give rise to a convex Arrhenius plot and possibly an optimum in *k*_cat_ that is not imposed by unfolding of the enzyme. As suggested by a referee of this paper, perhaps a nonzero activation heat capacity could be found for enzyme reactions where the substrate undergoes major conformational changes, such as in sterol or cyclooctatin biosynthesis.^39,40^ On the other hand, such reactions are very complex and involve many steps, which may obscure the picture.

In the case of the 1A53-2.5 designer enzyme, a convex Arrhenius plot with a temperature optimum of *k*_cat_/*K*_M_ at about 51 °C was observed, while the melting temperature of the enzyme was measured to be as high as 84 °C.^4^ This effect was suggested to originate from an optimum of *k*_cat_ caused by a negative activation heat capacity (Δ*C*_*p*_^‡^ < 0).^4,5^ However, as discussed above, earlier MD/EVB free energy calculations of the reaction barrier for the chemical step gave a perfectly linear Arrhenius plot with no indication of a negative value of Δ*C*_*p*_^‡^.^8^ To examine this result further, we have tried to calculate Δ*C*_*p*_^‡^, herein, by both the derivative and fluctuation formulas. The conclusion from the most accurate estimate (eq 2) is that Δ*C*_*p*_^‡^(TS – ES) is indeed very close to zero and certainly not of the negative magnitude that would be required if the optimum of *k*_cat_/*K*_M_ would be due to an optimum in *k*_cat_. Here, it should again be noted that the heat capacity calculation is also done for a fixed force field model of the TS, which is not exactly equal to the TS ensemble obtained from earlier MD/EVB simulations^8^ but a good approximation of it. In this sense, the direct calculation of activation free energies should clearly be more reliable than heat capacity estimates as far as judging whether the activation free energy is linear in temperature or not.

Nevertheless, we find that eq 2 can provide a very useful way of assessing possible heat capacity effects on ligand binding, which is of considerable interest also in drug design.^13^ In this case, it is presumably very difficult to directly obtain the temperature dependence of absolute binding free energies from computer simulations. Our results indicate that an accuracy of a few tenths of a kcal/mol/K for Δ*C*_*p*_^bind^ is attainable, and this can probably be pushed further with longer simulations. In the case of Kemp eliminase, we also find that Δ*C*_*p*_^bind^ is very small and does not either appear to be negative as would be needed to explain the *k*_cat_/*K*_M_ profile for 1A53-2.5 in terms of a Δ*C*_*p*_^bind^ = −0.3 kcal/mol/K. Hence, our best explanation for the curved Arrhenius plot in this case is still that there is a change of rate-limiting step at 308 K, and such a model is fully compatible with the calculated activation parameters for the chemical step.^8^
